# Supplementary material for: Scanning activity of elite football players in 11 vs. 11 match play: An eye-tracking analysis on the duration and visual information of scanning
Source: PLoS One. 2021 Aug 20;16(8):e0244118. doi: 10.1371/journal.pone.0244118 (PMC8378692; doi:10.1371/journal.pone.0244118)
Supplement: Attachment — Submitted filename: Response to Reviewers.docx [file pone.0244118.s004.docx]

Reviewer #1: I am happy to see the revisions made in the current version of the manuscript. I believe the paper is already much improved in its clarity, and the paper makes good logical sense. I have some concerns still (as outlined below), however I feel everything else in the manuscript is now much more strongly communicated. Well done so far to the authors.
- Overall, my comments below mostly relate to the operational definition of scanning information between movement and stop-point phases. I feel the current definition causes issues in interpretation of the findings, and often results in statements that could easily be falsely interpreted. The authors have offered to change their definition for movement phases to include information that is also included in the stop point. I will leave this to the authors to decide, however if they do wish to keep the current definition, they should be very careful about how the findings are interpreted and discussed.

**Thank you for this input. We decided to keep the definition as it is. However, we have made our interpretations of these findings as well as the following discussion much more cautious.**

- L240-241, can the authors please clarify this statement that these independent variables were not found to be relevant in providing further context for scanning information? It seems that this should be presented as a finding, rather than in the definition of independent variables. If a lack of difference in scanning information for these variables was found, this is still a finding, and should not be interpreted as providing a lack of context.

**Thank you for this input. I understand the confusion. We believed that control or pass, air or pitch, ball action, and the presence of fixations would add interesting information to the examination of scanning duration. Oppositely, we believed that playing phase and player-to-ball distance would provide enough context for the scanning information variable. There was never any plan to also include those four contextual variables for scanning duration.** **We decided to remove the entire sentence in order to not cause confusion.**

- L381-383, the players most often had zero players in the video frame during movement phases (Fig. 6). This is due to the categorisation of information to movement and stop point phases, right? If so, this statement should be clarified so as not to cause confusion for a reader.

**Thank you for this input! We added a sentence explaining that this result should be seen in light of our operationalization of the movement phase.**

- L391-393, I don’t know that the authors can state this, as this is clearly an artifact of the operational definition used in this study. I am fine with the authors using this definition, but it is my opinion that this type of summary statement cannot be used.

**We fully agree with your comment. We have removed that summary statement.**

- L444-445, for (b) for the same reason as (d) below, I don’t think this claim can be made with confidence. This is surely due to the definition used in the current study, and is not representative of the visual information that actually is available during both the movement and stop point phases? The separation of these variables is fine, but I do not believe that this sort of discussion (L544-554) can be made, knowing that it is entirely due to the definition and data analysis used.

**Thank you for this input. We changed the sentence from visual information to the number of teammates and opponents. We also made changes to the paragraph L544-554 according to your suggestions.**

- L446-447, for (d), this is only true because of the definition used in this study and the way data has been categorised. Again, there is information overlap between aspects of a scan, which is being ignored by the operational definition used in this case. In fact, if the definition would be reversed, such that the stop point phase did not include teammates and opponents that were detected in the movement phase, this finding would likely be opposite. Therefore, this sort of statement needs to be made carefully to ensure the actual finding is not overlooked. Rather than rationalise with the acceptance of the limitations, it would be better to not make these claims in the first place. Please amend.

**Thank you for this input. We agree an added “based on our operationalizations” at the beginning of the sentence.**

- L512-522, this is an interesting finding that is in line with previous research. However, it should be noted that part of this may again be due to the method of categorising scanning actions to start when the ball leaves the video frame. It is worth noting that the differences in classifying visual scanning actions between study methodologies, and the potential influence of this on findings.

**Great input! We added a sentence explaining that this result could be partly influenced by our operationalization which started measuring the duration at the moment the ball left the video frame.**

Reviewer #2: I want to commend the authors on their revision of the manuscript. They have done a great job revising the manuscript and have addressed most, if not all (apart from what is indicated below) of my previous comments and reservations.

My main concern (and associated advice) is that there are still occasions where the authors make statements that cannot be supported by the data collected and theory and analyses presented. For example, in the abstract, the authors conclude that the players detected more opponents than teammates (line 37). As also argued before, it is impossible with the presented methodology to establish what the participants actually saw or detected. Again, the authors equate having measured something being in the video-frame with it being detected (or not) or being seen (or not) by the players. What is established using the video is not necessarily the same as what was available in the full field of vision and something can be presented in the field of vision but not be seen by the athlete. So, my advice again is for the authors to stick to the data (what was and wasn’t on the video frame) rather than making interpretations (what was or wasn’t seen or detected by the players) – in particular when presenting the findings in the abstract (line 37) but also throughout, in the results, discussion (specifically, line 501/502; line 524-525 and paragraph from line 523) and conclusion (line 629).

**Thank you for this input! We changed all instances where we referred to seeing or detection. We believe the manuscript has benefitted greatly from this.**

Further, more detailed comments:

Line 239-240. It is announced that four independent variables were used to provide further context for scanning duration but that they were found NOT to be relevant. In what way were they not relevant and if they were not relevant, why are they presented and discussed? I was a bit confused by this statement.

**Thank you for this input. I understand the confusion. We believed that control or pass, air or pitch, ball action, and the presence of fixations would add interesting information to the examination of scanning duration. Oppositely, we believed that playing phase and player-to-ball distance would provide enough context for the scanning information variable. There was never any plan to also include those four contextual variables for scanning duration.** **We decided to remove the entire sentence in order to not cause confusion.**

Line 243 and beyond (whole paragraph and line 443 in discussion). To assist the reader to appreciate this paragraph, further clarification is needed as earlier it is stated that no scans were analysed where the player/participant themselves were in possession. This paragraph refers to possessions, but presumably of other players – NOT the participants themselves. This needs to be made more explicit and obvious for proper understanding of the methodology and findings. Mixed usage of players (i.e. other players) versus participants (i.e. the individuals who’s scans are collected and analysed) can cause this confusion.

**Great input! We added a sentence at the start of the first paragraph to emphasize this point.**

Line 356. As a methodological debate/discussion/argument will be developed in the discussion in regards to the ‘fixation’ cut-off of 120ms (as defined in the methods), it may be useful to emphasise throughout how fixations were determined by the current method. In the discussion reference is made to an alternative analysis with a 60ms fixation detection threshold. Authors may want to develop this argument a bit earlier as it is a very relevant finding of the current study that, based upon a fixation detection threshold of 120ms – and the mean scanning duration being 397ms. Future research will need to be aware of the need to scrutinise the currently ‘accepted’ fixation threshold of 120ms which has come about in research under lab conditions and quite often stationary (no head movements) tasks and frontal projections. Further references to literature making similar methodological arguments will be useful.

**Great input! We included a few sentences in the methods section in which we further explained the use of a 120 ms threshold while argued that lower thresholds, once accepted in the scientific community, might be better. Additionally, we added information that we used a 120 ms threshold when we presented the fixation results. Regarding your suggestion of adding references that supports this methodological argument, we were unable to find any. Could you please be specific regarding what paper(-s) you are referring to.**

Line 450. … therefor … can be deleted.

**Revised as suggested.**

Lines 439 and beyond. See also my comments above on definition of a fixation and presenting conclusions that are not grounded in evidence. Importantly, it is argued that fixations are absent – but there is also an argument that the fixation threshold may be too strict to pick up fixations. These two arguments partly contradict each other. Further, it is argued that the ‘absence’ of fixations evidences that they did not ‘see’ their opponents and teammates in ‘high definition’ (line 497/498); that information intake seldom (sometimes, never?) originates from ‘clear foveal images’; and that the findings merit understanding of scanning as a “blurred video”. There are quite a few unfounded or at best not discussed assumptions at play here about how human perception (and action) may function and these (non-apparent) assumptions are clearly not aligned with the explicit theoretical framework of ecological psychology that is introduced. One big assumptions from this framework is that human perception (and action) should NOT be equated (or compared!) to pictorial perception (Gibson, 1979). That is, the stimulus for visual perception is the optic array (the environment) – NOT the retinal image, so perception (and action) is NOT like watching images or videos. The explanation and understanding of visual perception as pictorial perception outlined in this paragraph is very confusing and unhelpful for proper interpretation of the findings and methodological and theoretical contribution of the paper. This paragraph, as well as the Practical applications paragraph (on lines 587/588 there is another reference to clear high definition pictures, which is problematic), need to be reworked to better align the argument to the theoretical framework of ecological psychology and to the methodological argument (and contribution) of the limitations of using a certain fixation threshold.

**Thank you for these comments**. (**1)** **Regarding the seemingly contradictory statements of fixations, we have to present the results from the accepted 120 ms threshold. As we mentioned in our last response, adopting a 60 ms threshold would result in approximately 18% of scans with fixations. Thus, we believe that we confidently can say that fixations are rare in scans, whilst adding the information that a lower threshold should perhaps be implemented in future research. (2) We removed or changed the wording “high definition”, “clear foveal images”, and “blurred video” to better align with our theoretical framework.**

Lines 597 and beyond. It wasn’t apparent to me how this advice could be directly derived from the findings presented. This may have been observed in these players, but does that merit that practice should be organised accordingly? And does that transfer to any level of practice – or only practice at the highest level? What about early learners? What if we want to teach the experts something new? The findings represent real world behaviour but that is not to say that it was optimal behaviour, simply because these were high-level athletes.

**This is a great input! We made several alterations to the practical applications for coaches, mostly related to the wording. We included words like “in line with our results” and “inspired by our results” instead of saying that it is directly derived. We also changed the wording from should to could and so on.**
