# Supplementary material for: Scanning activity of elite football players in 11 vs. 11 match play: An eye-tracking analysis on the duration and visual information of scanning
Source: PLoS One. 2021 Aug 20;16(8):e0244118. doi: 10.1371/journal.pone.0244118 (PMC8378692; doi:10.1371/journal.pone.0244118)
Supplement: Attachment — Submitted filename: Response to Reviewers.docx [file pone.0244118.s005.docx]

**Response to reviewers**

Throughout the authors should check that past tense is used when relevant. Below my pick-ups:

Line 79: Jordet suggested …
Line 121: As such, we aimed …
Line 123: the aim of this exploratory study was …
Line 243: operationalizations were made …
Line 455: initiation influenced …
Line 456: dinstance influenced …
Line 457: scans involved …
Line 458: scan revealed …
Line 564: players moved …
Line 564: until they arrived …
Line 565: they wished …

**Thank you for these observations! We changed the wording to the past tense.**


Lines 263/264 (and throughout): Please refer to fixation detection threshold throughout, so insert ‘fixation detection’ between ‘lower’ and ‘threshold’ here.
Line 264: It may be a while until a lower fixation detection threshold is accepted in the context of scanning. In light of this, consider replacing ‘accepted’ with ‘considered’.

**These are great comments. We added fixation detection throughout and changed the wording from accepted to considered.**

Lines 396-398: This sentence confused me. Maybe, for added clarity, add ‘in the video frame’ after ‘… more than seven teammates’ and after ‘… nine opponents’

**Great input! We added the words as suggested.**

Line 421: ‘while in defence’
Line 422/421: Consider rephrasing , eg: ‘… while in defence, no differences could be found between the amount of opponents and teammates in the video frame’.

**Thank you for this suggestion. We adopted it.**

Line 474: ‘the ball is being passed along the ground …’
Line 476: ‘the pass is made through the air …’

**Revised as suggested.**

Line 510-513: This reads like a left-over from a previous version. The reference to clear foveal information, as outline before, makes no sense from an ecological framework, and makes an implicit reference to perception of pictures. What’s more, whereas the manuscript now contributes to the readers understanding of the role of the fixation detection threshold in the determination of fixations, this sentence weakens that contributions (as you later explain in lines 516 – 523). **Thank you for this input. We fully agree and removed the entire sentence.**

Lines 652/653: As above, this sentence seems a left-over on the ‘seeing detail’ part (confusing perception and action with pictorial perception) and weakens the argument. I strongly suggest the authors rephrase or delete this very suggestive supposition that is neither based on theory nor finds any evidence in data presented.

**Thank you for this input. We removed the sentence an instead added two sentences that are more in line with the story of the paper, the results, and the ecological framework of perception-action coupling: *“This result can be partly explained by the adopted laboratory fixation detection threshold of 120 ms, which might be too high for unstable real-world research. However, it also implies that players, when performing scans during match-play, do not need to foveally fixate on surrounding information in order to obtain sufficient information for performing their football actions.”***

Line 517/518: As above, write ‘lower fixation detection threshold’ in full.

**Revised as suggested.**

Line 547: Insert ‘focus of’ so as to read: ‘… equivalent of focus of attention’

**Revised as suggested.**
